# World’s First Experience of the Low-Dose Radionuclide Inhalation Therapy in the Treatment of COVID-19-Associated Viral Pneumonia: Phase 1/2 Clinical Trial

**DOI:** 10.2174/1874471016666230307113045

**Published:** 2023-04-07

**Authors:** Peter Shegay, Alexey Leontyev, Denis Baranovskii, German Davydov, Marina Poluektova, Lyudmila Grivtsova, Vasily Petriev, Valeriy Stepanenko, Igor Gulidov, Valeriy Krylov, Svetlana Osadchaya, Vladimir Petrov, Maria Sedova, Mikhail Vekilyan, Olga Krasilnikova, Sergey Morozov, Sergey Ivanov, Ilya Klabukov, Andrey Kaprin

**Affiliations:** 1 Center of Innovative Radiological and Regenerative Technologies, National Medical Research Radiological Centre of the Ministry of Health of the Russian Federation, Obninsk, Russia;; 2 Internal Medicine Department, 24^th^ Moscow City State Hospital, Moscow, Russia;; 3 Department of Urology and Operative Nephrology, Peoples' Friendship University of Russia (RUDN University), Moscow, Russia;; 4 Research and Practical Center of Medical Radiology, Department of Health Care of Moscow, Moscow, Russia;; 5 Obninsk Institute for Nuclear Power Engineering, National Research Nuclear University MEPhI, Obninsk, Russia

**Keywords:** Low-dose, radionuclide therapy, COVID-19, sodium pertechnetate Tc^99m^, radiopharmaceuticals, pneumonia

## Abstract

**Objective:**

Previously, low-dose radiation therapy was used for pneumonia treatment. We aimed to investigate the safety and effectiveness of carbon nanoparticles labeled with Technetium isotope (^99m^Tc) in a form of ultradispersed aerosol in combination with standard COVID-19 therapy. The study was a randomized phase 1 and phase 2 clinical trial of low-dose radionuclide inhalation therapy for patients with COVID-19 related pneumonia.

**Methods:**

We enrolled 47 patients with confirmed COVID-19 infection and early laboratory signs of cytokine storm and randomized them into the Treatment and Control groups. We analyzed blood parameters reflecting the COVID-19 severity and inflammatory response.

**Results:**

Low-dose ^99m^Tc-labeled inhalation showed a minimal accumulation of radionuclide in lungs in healthy volunteers. We observed no significant differences between the groups before treatment in WBC-count, D-dimer, CRP, Ferritin or LDH levels. We found that Ferritin and LDH levels significantly raised after the 7th day follow-up only in the Control group (*p* < 0.0001 and *p* = 0.0005, respectively), while mean values of the same indicators did not change in patients in the Treatment group after the radionuclide treatment. D-dimer values also lowered in the radionuclide treated group, however, this effect was not statistically significant. Furthermore, we observed a significant decrease in CD19+ cell counts in patients of the radionuclide-treated group.

**Conclusion:**

Inhalation low-dose radionuclide therapy of ^99m^Tc aerosol affects the major prognostic indicators of COVID-19-related pneumonia restraining inflammatory response. Overall, we identified no evidence of major adverse events in the group receiving radionuclide.

In Memory of Prof. Vasily Petriev (1946-2023)

Prof. Vasily Petriev passed on January 8, 2023. He was a brilliant scientist in the field of radiopharmacy, a Habilitated Doctor of Biological Sciences, and one of the most experienced radiobiologists in Russia.

He was born in 1946 in Altai Krai, Russia, graduated from Lomonosov Moscow State University in 1970 majoring in radiochemistry.

Over 50 years of his career, Prof. Vasily Petriev developed numerous radiopharmaceuticals based on Technetium-99m, Rhenium-188, Lutetium-177, Actinium-225, and other isotopes. In 2021, he established the world’s first Nuclear Pharmacy for on-demand manufacturing of radiopharmaceuticals.

Prof. Vasily Petriev was distinguished by his devotion to science and persistent will to help humanity in its fight against cancer. His passing is an irreplaceable loss and tragedy for the whole scientific community.

## 1 INTRODUCTION

Explosive immune cell signaling caused by COVID-19 pneumonia is known as a cytokine storm and leads to life-threatening complications including respiratory distress syndrome [1]. Current therapeutic strategies are generally based on the prevention of inflammation-mediated lung injury by modulating the immune system of the host and, in particular, suppressing IL-cell signaling [2, 3]. Low-dose ionizing radiation can reduce inflammation *via* various mechanisms, including down regulated secretion of IL-beta-1 by activated macrophages and even could awake regeneration and anti-inflammatory cell signaling with TGF-beta-1 [4, 5]. Furthermore, Dhawan *et al*. (2020) suggested that a single irradiation dose of 0.3-0.5 Gy can possibly reduce the systemic inflammatory response [6].

Since low-dose radiation therapy was shown to have an anti-inflammatory effect, this method was successfully administered to treat bacterial and viral pneumonia in the pre-antibiotic era [7, 8]. Recent publications also hypothesize that low-dose radiation therapy (LDRT) may represent a promising method for the treatment of COVID-19-related pneumonia [5, 9, 10]. Preliminary research data showed LDRT as a feasible approach for the treatment of COVID-19 [11]. In the study by Hess *et al*. (2020), 80% of elderly patients with COVID-19 pneumonia were able to leave supplementary oxygen therapy at 1.5 days (mean) after low-dose whole-lung irradiation [12]. In the study by Ameri *et al*. (2020) low-dose whole lung irradiation with a single 0.5 to 1.0 Gy fraction led to fast initial improvement in SpO2 [13]. However, some authors still consider that radiotherapy should be confined to critically ill patients [6, 14]. Differing opinions regarding the potential efficacy of LDRT support the necessity of further clinical trials [15, 16].

In the majority of conducted studies, COVID-19 patients received external pulmonary irradiation. However, inhalation delivery is a promising technique for pneumonia treatment [17]. In our phase 1/ 2 clinical trial we investigated the safety and effectiveness of LDRT for patients with COVID-19-associated pneumonia. We provided LDRT in the form of inhalations containing the ultradispersed aerosol of ^99m^Tc-labeled carbon nanoparticles obtained by the TechnegasPlus generator.

## 2 MATERIALS AND METHODS

We conducted a single-center, non-randomized study at the National Medical Research Radiological Centre.

The phase 1 clinical trial was performed to determine the accumulated dose in human lungs during internal irradiation of ^99m^Tc aerosol in a group of healthy volunteers. Healthy volunteers without clinical and radiological signs of pneumonia were assigned to receive the inhalations with the aerosol. The calculation of the accumulated absorbed doses in the lungs and organs of volunteers from internal irradiation of ^99m^Tc aerosol was carried out according to the methodology of the Medical Internal Dose Committee (MIRD) [18].

Phase 2 was initiated after a ten-day safety observation period for all participants included in the phase 1 trial. We enrolled 47 patients suffering from COVID-19 pneumonia with early laboratory signs of the cytokine storm. Cytokine storm manifestation was defined in accordance with the interim guidelines for the prevention, diagnosis and treatment of new coronavirus infection (COVID-19) [1]. In particular, the criteria included increased serum ferritin levels > 300 ng/ml or a combination of two of the following indicators: blood platelets (PLT) ≤ 180×10^9^/l, leukocytes ≤ 3.0×10^9^/l, lymphopenia or a rapid decrease in PLT within 24 hours, increased activity of AST, serum triglycerides >156 mg/dL, decrease in blood fibrinogen ≤ 360 mg/dL.

**Fig. (1):**
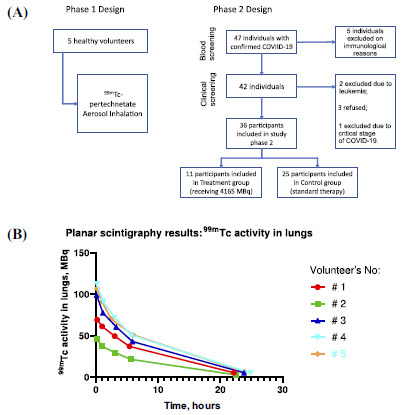
(**A**) Study profile; (**B**) Planar scintigraphy results for healthy volunteers: interpolation to one phase exponential decay.

We excluded 8 patients with the critical stage of COVID-19 (requiring treatment in intensive care unit), and oncological or autoimmune diseases from the study. Three patients refused further participation (Fig. **1**). Then, 11 patients were randomly assigned to the Treatment group. The control group consisted of 25 remaining participants.

During the phase 2 clinical trial, the Treatment group received inhalations of ^99m^Tc aerosol. Both groups also received standard therapy in accordance with the national temporary guidelines for prevention, diagnostics and treatment of COVID-19 (version 8) [19]. In accordance with the national guidelines from 03.09.2020, our patients did not receive glucocorticoid therapy.

The estimated sample size was calculated to be at least 10 participants in the Treatment and Control groups. Written informed consent was obtained from each participant. The study was approved by the Local Ethics Committee of A. Tsyb Medical Radiological Research Center - Branch of the National Medical Research Radiological Centre (Protocol No 510, September 17, 2020).

### 2.1 ^99m^Tc Aerosol Generation

^99m^Tc aerosol containing ^99m^Tc-labeled carbon nanoparticles (Na^99m^TcO_4_; the ^99m^Tc half-life is 6.04 hours) was obtained from generator ‘TechnegasPlus’ (Cyclomedica Australia Pty Ltd.). We generated 99mTc aerosol for each participant separately for immediate administration.

### 2.2 Dosimetry

Five healthy participants of the phase 1 clinical trial were assigned to estimate the accumulated internal absorbed doses in the lungs following the inhalations with the radioactive aerosol labeled by ^99m^Tc. We used the generator of carbon nanoparticles in the form of ^99m^Tc aerosol (TechnegasPlus, Cyclomedica Australia Pty Ltd). The mean initial radioactivity of the aerosol in the crucible ranged 887 ± 35 MBq. After inhalation, each of the volunteers underwent planar scintigraphy in the ‘whole body’ mode in the anterior and posterior front projections with contouring of the areas of interest using a two-detector rotary gamma camera combined with an X-ray computed tomography (Discovery 670 NM/CT, GE Healthcare), and with the recording of the gamma-quanta counts over the regions of interest. The following scanning parameters were used: speed of table deck movement 16 cm/min, matrix 256×1024, LEHR (low energy high resolution) collimators, photopeak 140.5 keV (^99m^Tc), discriminator window width ± 7.5%. GE Healthcare Xeleris v.3.0-3.1 workstations were used for image analysis. The used “body contour” mode provides registration of ^99m^Tc radiation at a distance of 3 cm from the contour of the surface of the phantom or the human body. Body weight, height, the age of the volunteer, the time interval between the preparation of the radioactive aerosol and the beginning of inhalation, the duration of inhalation, the number of inhales/exhales and the number of counts over the regions of interest were recorded. Planar scintigraphy was performed at 10 min, 1 h, 3 h, 6 h, 8 h and 24 h after inhalation.

To obtain the transition coefficients from the count rate over the region of interest, obtained from planar scintigraphy in the “whole body” mode in the anterior and posterior direct projections to the absolute radioactivity of ^99m^Tc in the region of interest, the special measurements were carried out using physical anthropomorphic phantoms. We used unified human anthropometric phantoms with different body weights [20], containing the standard activity of ^99m^Tc in a lung phantom located in the thoracic region. Three phantoms with masses of 55 kg, 77 kg and 100 kg were used. In the thoracic region of the phantoms used, phantoms of human lungs made of tissue-equivalent material with a density of 0.26 g/cm^3^ were located. The phantoms of the lungs have special tubes with a standard ^99m^Tc activity. To ensure uniform distribution of activity over the volume of the lungs, the possibility of uniform placement of ten tubes with a standard activity of ^99m^Tc was provided.

We obtained the functions of retention and excretion of ^99m^Tc activity in the regions of interest. After that, we calculated the time integrals of these functions, as well as the values of the specific fractions of absorbed energy from ^99m^Tc radiation using the Monte Carlo method, in order to determine the accumulated absorbed doses of internal radiation, in accordance with the general scheme of the Medical Internal Radiation Dose Committee (MIRD) [18].

### 2.3 COVID-19 Test

qPCR-RT detection of SARS-CoV-2 RNA was performed for all patients in phase 2 to confirm COVID-19.

### 2.4 Chest Computed Tomography (CT-Scan)

We performed chest CT for all participants in phase 1 and phase 2 on Somatom Sensation Open (Siemens) to indicate the stage of pneumonia on admission.

### 2.5 Planar Scintigraphy

In phase 1, the scintigraphy was performed 10 minutes, 1 hour, 3 hours, 6 hours, 8 hours, and 24 hours after the inhalation.

### 2.6 Blood Tests

In phase 2, blood tests were performed for all patients initially on the first day before any other procedures and on day 7 during the study.

Blood tests included quantitative measurements of the parameters with known greatest predictive significance for COVID-19 severity: D-dimer, White Blood Cells (WBC) and Platelets (PLT) counts, Lactate dehydrogenase (LDH), C-reactive protein (CRP), Ferritin. Additionally, we analyzed Immunogram of CD3^+^, CD4^+^, CD8^+^, and CD19^+^ cell counts for the patients in the Treatment group.

The assessment of biochemical parameters was carried out on an automatic analyzer AU 480 (Becman Coulter). The evaluation of immunological data (CD3^+^, CD4^+^, CD8^+^, and CD19^+^ cells) was carried out by the FACSCanto II flow cytometry. Flow cytometry data were processed in the FCS3.0 and Kaluza software applications (Becman Coulter).

### 2.7 Procedures Phase 1

In phase 1, five healthy volunteers underwent a single inhalation procedure of ^99m^Tc aerosol. The procedure consisted of a series of deep and slow inhalations through a special

system of tubes and filters with a 5-second delay between each breathing cycle. Inhalation was followed by planar scintigraphy after 10 min, 1 h, 3 h, 6 h, 8 h, and 24 h. The modes and geometry of measurements strictly corresponded to the modes and geometry of measurements on the phantoms (see section ‘Dosimetry’).

### 2.8 Procedures Phase 2

In phase 2, inhalation low-dose radionuclide therapy was carried out by inhalation of ^99m^Tc-labeled carbon nanoparticles, with pre-set radioactivity equal to 4165 MBq loaded *via* ^99m^Tc aerosol generator ‘TechnegasPlus’ (Cyclomedica Australia Pty Ltd.).

The treatment procedure consisted of a series of deep and slow inhalations with a 5-second delay between each breathing cycle.

Radionuclide therapy was carried out no later than one day after CT of the thoracic organs.

Clinical effectiveness was assessed on day 7 after the inhalation therapy during the period of hospitalization.

Methods and timing of assessment, registration, recording and analysis of medical indicators are described in Table **1**.

### 2.9 Outcomes

The primary outcome measures were values of Ferritin, LDH, D-dimer, CRP, WBC and PLT-counts after ^99m^Tc-pertechnetate Aerosol Inhalation.

The secondary outcome measures were counts of CD3^+^, CD4^+^, CD8^+^ and CD19^+^ cells after ^99m^Tc-pertechnetate Aerosol Inhalation.

### 2.10 Statistical Analysis

Statistical analysis was performed in GraphPad Prism 8.0 software (USA) using a Mixed-effects analysis with repeated measurements (analog ANOVA for the case of missed data on time points) with post hoc analysis according to Tukey's mean. Paired t-test or Wilcoxon signed ranks test were used for сomparison between lymphocytes subpopulation on Day 1 and Day 7 in the Treatment group. The level of statistical significance was taken as a *p*-value < 0.05.

## 3 RESULTS

Between October 15, 2020, and February 10, 2021, five healthy volunteers were included in phase 1, and 47 patients were screened for phase 2. However, 11 patients were excluded from the study phase 2 due to lymphoma, autoimmune diseases, patient refusal or critical stage of COVID-19 (Fig. **1A**). The trial recruited enough participants to evaluate whether inhalation low-dose radiotherapy had an effect on the main outcomes.

During the phase 1 clinical trial, no major adverse events were observed.

The baseline demographic characteristics of the participants were similar in the Treatment and Control groups (Table **2**).

### 3.1 Phase 1 Results

The radioactivity that entered the lungs of healthy volunteers following inhalation ranged from 5.5% to 12.4% of the initial activities of ^99m^Tc aerosol in the crucible. The values of internal pulmonary irradiation doses, normalized to the initial radioactivity of ^99m^Tc aerosol entering the lungs ranged from 0.0098 cGy/MBq to 0.012 cGy/MBq (on average, 0.011 ± 0.0009 cGy/MBq) (Table **2**).

Planar scintigraphy results confirmed decreasing the ^99m^Tc activity in the lungs (Fig. **1B**). One phase exponential decay showed a high correlation R2= [0,9895; 0,9993] with a half-life of ^99m^Tc activity in lungs ([4.5; 5.8] hours) close to the ^99m^Tc half-life (6.04 hours).

The residual radioactivity in the lungs of healthy volunteers following inhalation ranged from 5.5% to 12.4% of the initial values for aerosol (Table **2**). Accumulated doses ranged between 0.45 cGy and 1.24 cGy (mean, 0.94 cGy).

### 3.2 Phase 2 Results

COVID-19 was identified by positive qPCR for SARS-CoV-2 for all patients included in Phase 2 clinical trial. The Control and Treatment groups did not differ significantly according to the grade of viral pneumonia indicated by a CT thorax scan on admission.

The dynamics of the parameters of the patients who received inhalation low-dose radionuclide therapy are presented in Table **3**.

We observed no significant differences in WBC-count, D-dimer, CRP, Ferritin, or LDH levels between Treatment and Control groups on day one. However, PLT-count was found to be initially lower in the Control group (*p* < 0.0001).

**Fig. (2):**
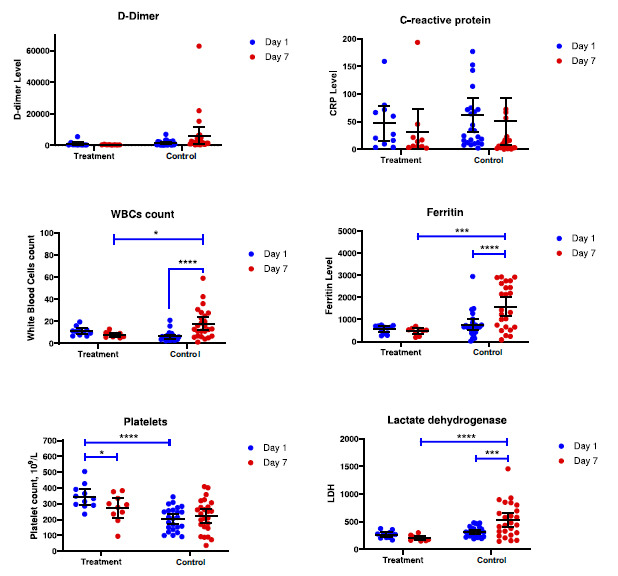
The D-dimer, C-reactive protein, WBCs, Ferritin, Platelets count and LDH. Two-way ANOVA with RM (mixed analysis). The error bars indicate the 95% CI of the Mean and the spots indicated the individual blood parameters. Only *p*-values for significant differences are shown on the figure.

On the 7th day of follow-up, the levels of CRP and D-dimer did not change significantly either in the Treatment or in the Control group (Fig. **2**). The Ferritin and LDH significantly raised after the follow-up in the Control group (*p* = 0.0002 and *p* < 0.0001, respectively), however, keeping the stable values in patients after the radionuclide treatment. Furthermore, both Ferritin and LDH values on day 7 were found to be lower in the Treatment than in the Control group (*p* = 0.0002 and *p* < 0.0001). D-dimer values also lowered in the radionuclide treated group, however, this effect was not statistically significant.

Platelet counts decreased in the Treatment group only, however, this result was significant at the *p* = 0.0469 level. At the same time, the PLT-count was found to be initially lower in the Control group (*p* < 0.0001).

The WBCs count increased in the Control group on day 7 (*p* < 0.0001), in contrast, the Treatment group had no significant differences. On day 7, there were differences between groups (*p* = 0.0105), while initially there were no differences.

**Fig. (3):**
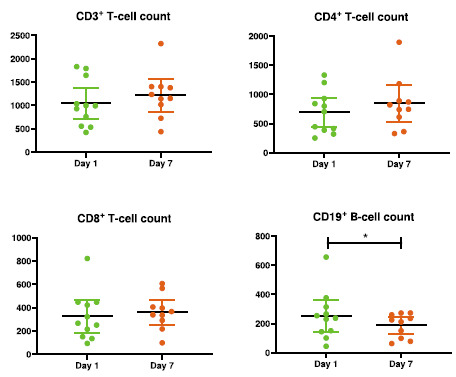
Immunogram of the Treatment group: CD3^+^, CD4^+^, CD8^+^, and CD19^+^ cell count presence. The error bars indicate the 95% CI of the Mean and the spots indicated the individual blood parameters. Only *p*-values for significant differences are shown in the figures.

In the Treatment group, the absolute value of CD19^+^ cell count on 7th day of follow-up was significantly decreased compared to day 1 (*p* = 0.043). At the same time CD3^+^, CD4^+^, and CD8^+^ have had no differences (Fig. **3**).

## 4 DISCUSSION

It was found that after inhalation of ^99m^Tc aerosol, the fraction of the initial radioactivity of ^99m^Tc that entered the lungs of volunteers varied in the range from 5.5% to 12.4% (on average 9.72%) in relation to the initial crucible radioactivity (Table **2**). Consequently, the intensity of the radioactivity intake varied from person to person, most likely, depending on the individual characteristics of the person, lungs ventilation capacity, body weights, depth and frequency of respirations during inhalation. The values for internal pulmonary irradiation doses were in the range from 0.0098 cGy/MBq to 0.012 cGy/MBq (mean, 0.011 cGy/MBq). Being normalized to the initial radioactivity, observed values of the average absorbed doses are very close to that for a standard model for adults [21].

More than 96% of inhaled ^99m^Tc nanoparticles accumulated in the pulmonary parenchyma within 6-48 hours after completion of inhalation and only about 3-4% of injected radioactivity accumulated in the urinary bladder [21]. According to the current data, accumulation of radiopharmaceuticals in the liver or spleen parenchyma was not observed [22].

Inflammation can further increase local blood supply, and cause edema and ventilation disturbances, reducing the absorption of nanoparticles and consequently the value of the accumulated dose in the lungs.

The absence of differences among CRP and D-dimer values may be caused by the short-term observations or a small number of patients in both groups. At the same time, these results can be explained by the lower clinical significance of the selected parameters despite their known correlation with COVID-19 severity according to previous studies [23].

Increasing LDH and Ferritin levels, observed in the control group, are known to be among the leading changes in blood parameters in patients with severe COVID-19 infection [24-26]. Rising ferritin level in COVID-19 patients also correlates with pneumonia severity, poor disease outcome and likelihood of mortality [24]. Low-dose radionuclide inhalation therapy for COVID-19 patients at least demonstrated stabilizing effect on WBCs count, Ferritin, and LDH levels, thus predicting a favorable disease outcome *versus* the standard therapy. Taken together those findings further support the conclusion that low-dose radionuclide inhalation therapy had clear benefits restraining an inflammatory response and preventing a cytokine storm.

Decreased platelets count after the inhalation therapy may be presumably a result of radiotherapeutic influence on PLT-synthesis in the lungs.

The observed recession of CD19^+^ cell count was clearly an unanticipated finding, but possibly defining an immunological impact of aerosol administration. CD19^+^ cells count was shown to be significantly less in severe COVID-19 patients [27, 28].

Presumably, the 7 days observation period is insufficient for assessing statistically significant changes in the majority of blood parameters but enough to detect early differences in sensitive indicators of cytokine storm and quantitative varieties in cell subpopulations [29].

## CONCLUSION

Inhalation low-dose radionuclide therapy with ^99m^Tc aerosol appeared to be safe according to the phase 1 clinical trial. We observed a positive effect on the key prognostic indicators of COVID-19 severity and it potentially could prevent the development of a cytokine storm. Patients benefited from the therapy according to their LDH and Ferritin levels after one-week after treatment in comparison with the Control group. However, our clinical trial was limited by the number of patients enrolled in short-term single center study. Further extended clinical trials are required to establish the effectiveness of low-dose therapy in COVID-19 pneumonia. Novel protocols including therapy with increased doses or repeated courses of inhalation may improve an observable effect of the treatment.

## Figures and Tables

**Table 1:** Methods and timing of assessment, registration, recording and analysis of medical indicators.

**Methods of Assessment**	**Terms**
Chest CT	Before treatment, on day 7 after radionuclide therapy
Blood tests	- Before treatment; - on day 7 after radionuclide therapy
Immunogram (CD3^+^, CD4^+^, CD8^+^, and CD19^+^ cell counts) *was performed for the treatment group only*	- before treatment; on day 7 after radionuclide therapy

**Table 2:** Results of cumulative internal lung doses in healthy volunteers after inhalation of ^99m^Tc.

**S. No.**	**Age**	**Activity of ^99m^Tc in Crucible, MBq**	**The Initial ^99m^Tс Activity Entered to the Lungs, MBq**	**Accumulated Dose in the Lungs, cGy**	**Accumulated Dose in Lungs Per Unit of Initial Inhaled ^99m^Tс** **Radioactivity in Lungs, cGy/MBq**	**The Required Activity of ^99m^Tс in the Crucible to Create a Lung Dose of 10 cGy, GBq**
1	61	897	69.3 (7.7%)	0.81	0.012	11.1
2	62	843	46.1 (5.5%)	0.45	0.0098	18.5
3	64	917	99.1 (10.8%)	0.99	0.010	19.1
4	67	921	112.8 (12.2%)	1.24	0.011	7.1
5	55	859	106.8 (12.4%)	1.20	0.011	7.1
Mean (SD)	-	887 (35)	86.8 (9.8%) (28.2)	0.94 (0.32)	0.011 (0.0009)	12.6 (5.9)

**Table 3:** Dynamics of clinical blood parameters in COVID-19 patients.

**S. No.**	**-**	**COVID-19 Patients with Low-Dose** Inhalation Therapy and Standard Therapy* Treatment Group		COVID-19 Patients with Standard Therapy* **Control Group**	
		**Day 1** ***(Before Therapy)***	**Day 7**	**Day 1** ***(Before Therapy)***	**Day 7**
1	Number of patients, n	11		25	
2	Age 18-64 years 65+	62.4 (8.9) [49÷80] 7 (63.6%) 4 (36.4%)		61.9 (10.2) [38÷76] 12 (48%) 13 (52%)	
3	Gender Female Male	7 4		13 12	
4	CT thorax on admission (Grade of viral pneumonia)	Grade 1 = 5 (46%) Grade 2 = 4 (36%) Grade 3 = 1 (9%) Grade 4 = 1 (9%)	n/a	Grade 1 = 11 (44%) Grade 2 = 11 (44%) Grade 3 = 1 (4%) Grade 4 = 2 (8%)	n/a
**Coagulogram**					
5	D-dimer (ng/ml)	995.3 (1651.9)	363.8 (156.6)	1347.9 (1600.1)	6224.4 (13060.7)
**Clinical Blood Analysis**					
6	WBC (10**^9^**/L)	11.2 (3.9)	8.0 (2.5)	6.4 (4.3)	18.0 (13.6)
7	Platelets counts (10**^9^**/L)	344 (75)	273 (86)	203 (74)	223 (102)
**Blood Biochemistry**					
8	Lactate dehydrogenase (LDH) (U/L)	266.5 (63.4)	204.0 (47.1)	317.8 (92.4)	527.1 (313.5)
9	C-reactive protein (mg/L)	47.1 (46.9)	31.4 (58.4)	61.6 (74.6)	51.1 (100.1)
10	Ferritin (ng/ml)	568 (181)	471 (164)	762 (581)	1581 (999)
**Immunogram**					
11	СD3^+^ cell count (10^9^/L)	1046 (503)	1223 (787)	n/a	n/a
12	СD4^+^ cell count (10^9^/L)	694 (364)	845 (447)	n/a	n/a
13	СD8^+^ cell count (10^9^/L)	325 (206)	363 (149)	n/a	n/a
14	СD 19^+^ cell count (10^9^/L)	252 (165)	187 (82)	n/a	n/a

**Note:** *Standard therapy was provided in accordance with Temporary Guidelines “Prevention, diagnosis and treatment of new coronavirus infection (COVID-19)” in Version 8, actual for September 2020 and included Favipiravir (in weight dependent doses), acetylcysteine and thromboprophylaxis. Data presented as: Mean (Standard Deviation).

## Data Availability

The data and supportive information are available within the article.

## LIST OF ABBREVIATIONS

CRP: C-Reactive Protein
LDRT: Low-Dose Radiation Therapy
MIRD: Medical Internal Dose Committee
PLT: Platelets

## STANDARDS OF REPORTING

CONSORT guidelines were followed.
