# Supplementary material for: World’s First Experience of the Low-Dose Radionuclide Inhalation Therapy in the Treatment of COVID-19-Associated Viral Pneumonia: Phase 1/2 Clinical Trial
Source: Curr Radiopharm. 2023 Apr 7;16(3):243–52. doi: 10.2174/1874471016666230307113045 (PMC11851150; doi:10.2174/1874471016666230307113045)
Supplement: Supplementary file 1 — Supplementary material is available on the publisher’s website along with the published article. [file CRP-16-3-243_SD1.pdf]

SUPPLEMENTARY MATERIALS

World's First Experience of the Low-Dose Radionuclide Inhalation Therapy in the Treatment of COVID-19-Associated Viral Pneumonia: Phase 1/2 Clinical Trial

Peter Shegay[1], Alexey Leontiev[1], Denis Baranovskii[1,2,3], German Davydov[1], Marina Poluektova[1], Lyudmila Grivtsova[1], Vasily Petriev[1], Valeriy Stepanenko[1], Igor Gulidov[1], Valeriy Krylov[1], Svetlana Osadchaya[1], Vladimir Petrov[1], Maria Sedova[1], Mikhail Vekilyan[1], Olga Krasilnikova[1], Sergey Morozov[4], Sergey Ivanov[1], Iiya I.D[1,3,5,*], and Andrey Kaprin[1,3]

[1]National Medical Research Radiological Center of the Ministry of Health of the Russian Federation, Obninsk, Russia; [2]24th Moscow City State Hospital, Moscow, Russia; [3]Peoples' Friendship University of Russia (RUDN University), Moscow, Russia; [4]Research and Practical Center of Medical Radiology, Department of Health Care of Moscow, Moscow, Russia; [5]Obninsk Institute for Nuclear Power Engineering, National Research Nuclear University MEPhI, Obninsk, Russia

CONSORT CHECKLIST

Section/Topic	Item No	Checklist item	Reported on page No
		**Title and abstract**	
	1a	Identification as a randomised trial in the title	This was a non-randomised controlled study
	1b	Structured summary of trial design, methods, results, and conclusions (for specific guidance see CONSORT for abstracts)	1
		**Introduction**	
Background and objectives	2a	Scientific background and explanation of rationale	1-2
	2b	Specific objectives or hypotheses	2
		**Methods**	
Trial design	3a	Description of trial design (such as parallel, factorial) including allocation ratio	2
	3b	Important changes to methods after trial commencement (such as eligibility criteria), with reasons	2
Participants	4a	Eligibility criteria for participants	2
	4b	Settings and locations where the data were collected	2
Interventions	5	The interventions for each group with sufficient details to allow replication, including how and when they were actually administered	2-3
Outcomes	6a	Completely defined pre-specified primary and secondary outcome measures, including how and when they were assessed	3
	6b	Any changes to trial outcomes after the trial commenced, with reasons	N/A
Sample size	7a	How sample size was determined	2
	7b	When applicable, explanation of any interim analyses and stopping guidelines	N/A
Randomisation:			This was a non-randomised controlled study
Sequence generation	8a	Method used to generate the random allocation sequence	This was a non-

			randomised controlled study
	8b	Type of randomisation; details of any restriction (such as blocking and block size)	This was a non-randomised controlled study
Allocation conceal-ment mech-anism	9	Mechanism used to implement the random allocation sequence (such as sequentially numbered containers), describing any steps taken to conceal the sequence until interventions were assigned	This was a non-randomised controlled study
Implementation	10	Who generated the random allocation sequence, who enrolled participants, and who assigned participants to interventions	This was a non-randomised controlled study
Blinding	11a	If done, who was blinded after assignment to interventions (for example, participants, care providers, those assessing outcomes) and how	This was a non-randomised controlled study
	11b	If relevant, description of the similarity of interventions	N/A
Statistical methods	12a	Statistical methods used to compare groups for primary and secondary outcomes	3
	12b	Methods for additional analyses, such as subgroup analyses and adjusted analyses	3
		**Results**	
Participant flow (a diagram is strongly recommended)	13a	For each group, the numbers of participants who were randomly assigned, received intended treatment, and were analysed for the primary outcome	4
	13b	For each group, losses and exclusions after randomisation, together with reasons	This was a non-randomised controlled study
Recruitment	14a	Dates defining the periods of recruitment and follow-up	3
	14b	Why the trial ended or was stopped	4
Baseline data	15	A table showing baseline demographic and clinical characteristics for each group	3
Numbers analysed	16	For each group, number of participants (denominator) included in each analysis and whether the analysis was by original assigned groups	4
Outcomes and esti-mation	17a	For each primary and secondary outcome, results for each group, and the estimated effect size and its precision (such as 95% confidence interval)	4
	17b	For binary outcomes, presentation of both absolute and relative effect sizes is recommended	4
Ancillary analyses	18	Results of any other analyses performed, including subgroup analyses and adjusted analyses, distinguishing pre-specified from exploratory	3-5
Harms	19	All important harms or unintended effects in each group (for specific guidance see CONSORT for harms)	4-5
		**Discussion**	
Limitations	20	Trial limitations, addressing sources of potential bias, imprecision, and, if relevant, multiplicity of analyses	5
Generalisability	21	Generalisability (external validity, applicability) of the trial findings	5
Interpretation	22	Interpretation consistent with results, balancing benefits and harms, and considering other relevant evidence	5
		**Other information**	
Registration	23	Registration number and name of trial registry	5
Protocol	24	Where the full trial protocol can be accessed, if available	5
Funding	25	Sources of funding and other support (such as supply of drugs), role of funders	5
